# Modelling Singapore COVID-19 pandemic with a SEIR multiplex network model

**DOI:** 10.1038/s41598-021-89515-7

**Published:** 2021-05-12

**Authors:** N. N. Chung, L. Y. Chew

**Affiliations:** 1 Centre for University Core, Singapore University of Social Sciences, Singapore, 599494 Singapore; 2 School of Physical & Mathematical Sciences, Nanyang Technological University, Singapore, 637371 Singapore; 3 Data Science & Artificial Intelligence Research Centre, Nanyang Technological University, Singapore, 639798 Singapore; 4 Complexity Institute, Nanyang Technological University, Singapore, 637723 Singapore

**Keywords:** Applied mathematics, Complex networks

## Abstract

In this paper, we have implemented a large-scale agent-based model to study the outbreak of coronavirus infectious diseases (COVID-19) in Singapore, taking into account complex human interaction pattern. In particular, the concept of multiplex network is utilized to differentiate between social interactions that happen in households and workplaces. In addition, weak interactions among crowds, transient interactions within social gatherings, and dense human contact between foreign workers in dormitories are also taken into consideration. Such a categorization in terms of a multiplex of social network connections together with the Susceptible-Exposed-Infectious-Removed (SEIR) epidemic model have enabled a more precise study of the feasibility and efficacy of control measures such as social distancing, work from home, and lockdown, at different moments and stages of the pandemics. Using this model, we study an epidemic outbreak that occurs within densely populated residential areas in Singapore. Our simulations show that residents in densely populated areas could be infected easily, even though they constitute a very small fraction of the whole population. Once infection begins in these areas, disease spreading is uncontrollable if appropriate control measures are not implemented.

## Introduction

The 2019 coronavirus outbreak has become a pandemic. For the first time in human history, we observe a global concerted effort that tries to stem the spread of the epidemic with the semi-closing of national borders and grounding of international flights. Each country has its unique strategies to contain the virus in their territories. This ranges from complete lockdown in China and the herd immunity cum gradual intervention approach in Sweden^1^. While the most direct approach to arrest the virus is to create a vaccine against it, the lead time to vaccine development is long relative to the virulence of the disease that the best short-term strategy is social exclusion.

The coronavirus spreads through the simple mechanism of human-to-human contact. This is the reason why governments around the world implement lockdown and restrict social interactions. Ideally, if we could isolate everyone, there would be no room for the virus to survive. But this is socially impossible for family members. In addition, key economic activities still need to go on for the whole society to function, and this requires social interactions from workers providing essential services. There are two practical means to prevent the spreading of the virus through such a social route. One is to quarantine all infected individuals and also their contacts^2^. This necessitates, however, all infected individuals to be identified immediately once they become infectious. But there is always a delay in such identification, and worst of all, some infected individuals are asymptomatic. The capability to carry out large-scale swap test has thus become important as it reduces not only the delay from disease onset to isolation, but also the number of undetected spreaders. The second approach is social distancing whose implementation has already severely disrupted the economies of many countries.

In order to properly manage the social-economic impact of the COVID-19 pandemic, governments would need a clear rationalization on the right policies to undertake. There are questions on whether lockdown should be implemented, like the case of Sweden versus its Scandinavian neighbors. And if lockdown were to be lifted, how gradual should we relax the restriction order? The means to address these questions together with the consequential advisories to the governments have been well served by mathematical and computational models. For example, a meta-population mobility model based on microscopic Markov chain approach^3^ has been used to forecast the number of COVID-19 cases in each municipality of Spain. The outcome of the simulation had informed the Catalonian government ahead of time whether any Spanish region had exceeded its treatment capacity which led to a quick implementation of intervention measures that addressed the shortages. Meta-population based global epidemic and mobility model was also employed in another context to assess the efficacy of travel restrictions on the spread of COVID-19 at both the national and international level^4^. The results in this work showed that travel ban had mainly delayed the progression of the disease, with mitigation best achieved through transmission reduction intervention. In another modeling effort^5^, the effects of reopening schools as part of exit strategies of lockdown was examined. It found that a gradual reopening of schools is necessary not to overwhelm the existing healthcare system or to cause a new second wave of epidemic transmission. There are also a few studies which look into the transmission of COVID-19 in Singapore^6,7^. All these models use real-world data to constantly calibrate their computations to drive the simulated scenarios close to reality and near to the ground truth. And their insights guide governments in their making of policies and decisions. In fact, the literature of epidemiological analysis is growing fast amid the COVID-19 pandemics, a review of the most relevant existing literature can be found in Ref.^8^.

### Model

In this paper, we model the spreading of COVID-19 pandemics using the Susceptible-Exposed-Infectious-Removed (SEIR) model. Unlike the modified version of SEIR model used currently by a few research groups to simulate COVID-19 spreading^9,10^, our approach exploits both multiplex^11^ and temporal networks^12^ in conjunction with the SEIR model. Multiplex network had been used in Ref.^13^ to study how the allocation of resources in a social support layer affects the spreading of virus in the physical contact layer. There are also prior research that combine SIR or SIS model with multiplex networks to investigate the effects of immunization^14^, dynamical interactions between viral agents^15^, emergence of epidemic phases^16^, and contagion dynamics^17^. Recent research efforts on SEIR model on multiplex network have explored into the spread of rumors^18^, and the transmission of COVID-19 in and between different layers of transportation networks^19^.

The purpose and aim of our SEIR multiplex network model is to emulate the different forms of real-world social interactions in order to examine the effectiveness of various intervention strategies against the spread of COVID-19. The ability to tweak social interactions in mathematical models is crucial to find ways to curb the accelerating tides of epidemic spreading, in addition to giving insights on appropriate exit strategies from lockdown measures as the pandemic is entering the deceleration phase^20^. The main application of our model will be on the evaluation of the pandemic conditions in Singapore.

#### SEIR model

In SEIR model, individuals are classified into four infection stages, namely susceptible, exposed, infectious, and removed. All individuals in the population are assumed to be susceptible to the virus before the pandemic begins. The model starts when the first infectious individual is imported into the population of susceptible individuals. Note that this imported individual is chosen at random from the population. Following this, *M*(*t*) individuals are selected uniformly at random at each simulated day *t* to model the *M*(*t*) number of imported cases. As the focus of our study is on the pandemic in Singapore, the information of these imported cases was obtained from the Singapore Ministry of Health webpage^21^.

After a susceptible individual comes into contact with an infectious individual, the susceptible individual becomes exposed with a probability *p*. Note that it takes $T_e$ days on average before an exposed individual becomes infectious. Here, the exposure period is assumed to be gamma-distributed with a mean of $T_e$ days. Once the status of an individual is changed to infectious, it spreads the virus to each of its susceptible contact with a probability of *p*. Note that each individual can become infectious for different number of days depending on when they show the symptoms of infection and the time span from disease onset to social isolation. In addition, the infectious status of an otherwise asymptomatic individual is assumed to cease only when the individual has recovered. The infectious period is thus assumed to be gamma-distributed with a mean of $T_i$ days. Once recovered, an individual is no longer susceptible to the disease and can no longer become infectious.

#### Social interactions

Real-world social interaction is often too complex to be represented by ideal complex network models such as the Erdős-Rényi random network and the scale-free network. Here, we build a multiplex network^11^ which is composed of multiple overlapping networks that describe the various types of social connections between agents to study the dynamics of the epidemic outbreak. Specifically, our multiplex network consists of a household network, a dormitory network, a workplace network, a temporal crowd network, and a temporal social gathering network. Note that this list of networks is not exhaustive. In principle, any community which is socially connected in a significantly different way and is not in the existing list of communities in the model should be added as a separate layer in the multiplex network. For example, a slum network can be included in our model for cities with a slum neighborhood.

Let us now go into the details of each of our social network. We have a household network in our model which captures the social interactions among family members within a household. It is represented by a complex network with a community structure. Agents that belong to the same household (or community) are more densely connected internally than with the other agents within the household network. The size of the household is assumed to be Poisson distributed with an average of $S_h$ members. On the other hand, social interactions within worker dormitories is modelled separately by a dormitory network as dormitories house a much larger number of residents compared to normal households. In addition, individuals within dormitories possess a larger number of social connections with other residents who stay in the same dormitory. Note that our special interest in worker dormitories stems from the occurrence of a virulent spread of COVID-19 within the dormitory clusters within Singapore.

We model the social contact within and between workplaces by a workplace network. In Singapore, $40 \%$ of the population are employed and work in a workplace. Each workplace is modelled as a community in the workplace network. The size of a workplace is assumed, however, to be gamma-distributed so as to include workplaces of extremely large size. Note that for the sake of simplicity, we have modeled social interactions within schools in the same way as that of a workplace.

The social interactions within public spaces, such as the public transportation system and markets, is very different from fixed households or in the workplaces. It involves short-term interactions among random groups of individuals each day. In our model, we select $f_c$ groups of $N_c$ agents on average uniformly and randomly on each simulated day to form $f_c$ fully-connected temporal networks. The spreading probability $p_c$ within each of these crowds of agents is assumed to be smaller than those of the household or workplace networks. Specifically, it is $p_c = 0.1p$. In addition, we model social gatherings such as religious services, academic conferences, and large-scale dinner events in a similar fashion albeit with a different topology for their social interactions. On each simulated day, $f_g$ groups with an average size of $N_g$ agents are formed uniformly at random with the topology of scale-free temporal networks of average degree $k_g$ as their mode of transient social interactions. We assume the virus spreads with probability *p* in these social gathering networks.

### Combining SEIR model and multiplex network

Mathematically, the combined model can be represented by the following equations:

$$\begin{aligned} {\dot{S}}_i(t)= & {} - p \, S_i(t) \sum _{j=1}^N A_{ij} I_j(t) - p \, S_i(t) \sum _{j=1}^N A^g_{ij}(t) I_j(t) - p_c \, S_i(t) \sum _{j=1}^N A^c_{ij}(t) I_j(t) \,,\\ {\dot{E}}_i(t)= & {} p \, S_i(t) \sum _{j=1}^N A_{ij} I_j(t) + p \, S_i(t) \sum _{j=1}^N A^g_{ij}(t) I_j(t) + p_c \, S_i(t) \sum _{j=1}^N A^c_{ij}(t) I_j(t) -\sigma E_i(t) \,,\\ {\dot{I}}_i(t)= & {} \sigma E_i(t) - \gamma I_i(t) \,,\\ {\dot{R}}_i(t)= & {} \gamma I_i(t)\,, \end{aligned}$$

where *N* is the total number of nodes or individuals in the network. Here, $S_i(t)$, $E_i(t)$, $I_i(t)$ and $R_i(t)$ are the probability for the individual *i* to be susceptible, exposed, infectious and recovered at time *t* respectively. The rate at which exposed individuals become infectious is $\sigma$ while the rate at which infected individuals recover is denoted by $\gamma$. *A* is the adjacency matrix of the overall network, which incorporates social interactions in households, workplaces, and dormitories; $A^g(t)$ is the adjacency matirx of the temporal social gathering network; and $A^c(t)$ is the adjacency matirx of the temporal crowd network.

## Results

**Figure 1:**
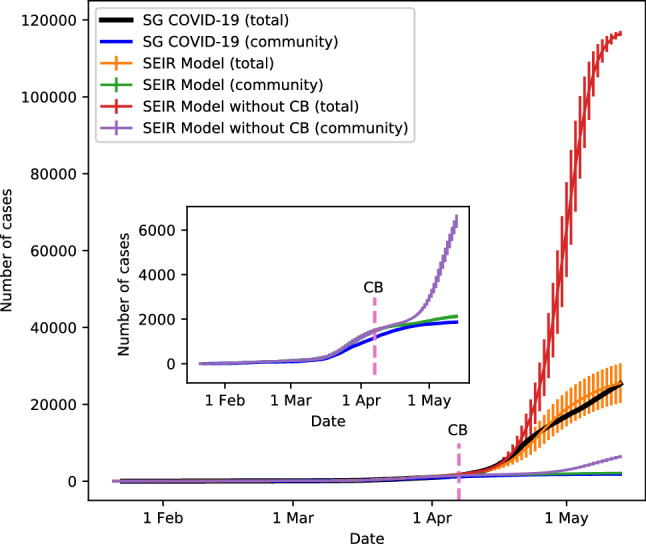
Epidemic curves for the spreading of COVID-19 in Singapore. The curves are shown for the real data, and for the simulated dynamics with and without implementation of the Circuit Breaker (CB) measures. The inset shows an enlarged view of the spreading of COVID-19 within Singapore’s community. Standard deviation is shown as vertical line for the simulated dynamics. Note that the dashed vertical lines in both plots mark the beginning of CB.

The outcome of our simulation is illustrated in Fig. 1. The simulation results with CB show good correspondence with the real data for both the total number of reported cases as well as the reported cases for just the community spread of COVID-19 (i.e., cases that exclude infections within dormitories). In contrast, a SEIR model with social contacts modelled by a simple Erdős-Rényi or scale-free random network cannot adequately capture the spreading dynamics of COVID-19 in Singapore (see the Supplementary materials for details). We have also looked at the number of unlinked cases. As of 13 May 2020, Singapore reported around 570 cases of COVID-19 with unknown sources of infection. In our model, unlinked cases arise from infections within a crowd network as well as by transmissions from individuals with mild or no symptom. At the end of the simulated period, we observed 314 infected cases from the crowd network. By assuming that an undetected individual is able to spread the virus for 7 days or more, we classified 137 cases as undetected at the end of the simulated period. Note that such cases have not happened in the dormitory network because the government has been actively screening COVID-19 infection in dormitories’ workers since the CB. In our simulation, we found another 290 infections due to these undetected cases on average. Assuming that some of these cases can be back-traced eventually through routine screening to asymptomatic individuals, our simulation would have uncovered 314 to 604 cases with unknown sources of infection.

Our model allows us to simulate the scenario if the government has not implemented the circuit breaker and has not revamped the living conditions of the foreign workers. Under this scenario, our results show an exponential rise in the total number of cases. Note that the number of cases will be even more if not for the maximum capacity of 110, 000 dormitory workers we have fixed in our simulation. This explains the appearance of a saturation in the curve for the total number of cases without CB. Thus, the CB has successfully contained by much more than 4 times the spread of COVID-19, and it has correspondingly reduced and prevented the stress on the medical facilities due to the pandemic.

**Figure 2:**
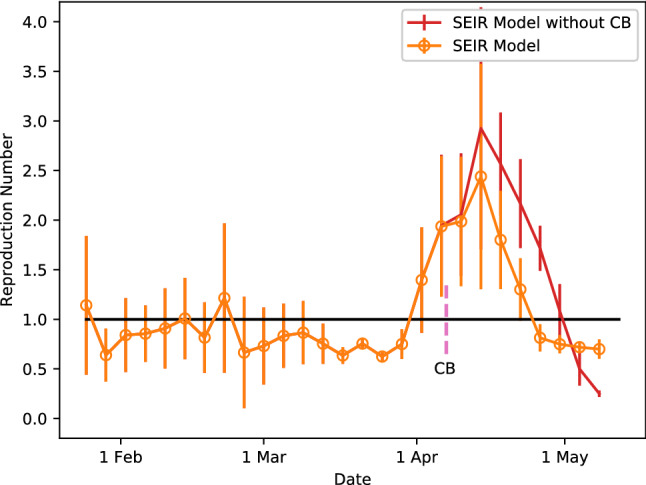
The dynamical evolution of the reproduction number $R_e$ derived from the SEIR multiplex model with and without Circuit Breaker (CB) measures. Note that the dashed vertical line marks the beginning of CB.

Figure 2 illustrates the progression of the pandemic in Singapore through the dynamics of $R_e$. We observe that $R_e$ predicts the emergence of three waves of enhanced COVID-19 transmission when $R_e$ becomes larger than one. The first wave happened on 14 Feb 2020 according to Fig. 2. It corresponds to an infection cluster at the church of the Grace Assembly of God with its first reported case on 12 Feb 2020 (which is also linked to reported cases from the Life Church and Missions Singapore)^22^. There are a total of 33 infected individuals in this cluster. The second wave began on 22 Feb 2020 (see Fig. 2). It is related to another infection cluster due to a private function at SAFRA Jurong with its first reported case on 27 Feb 2020. The total number of infected individuals for this cluster is 48. These two waves of COVID-19 transmission are in fact exceedingly small relative to the third wave which was started on 29 Mar 2020 based on the $R_e$ illustrated in Fig. 2. The third wave is a very large wave caused by the circulation of COVID-19 within the dormitories of the foreign workers. Although the first reported case was published on 5 April 2020, its presence could already be preempted from the reported case of infection within the construction site in the Raffles Place area on 3 April 2020. The total number of infected foreign workers in the dormitories is about 23, 000 by current count, and the cluster is still growing. The time delay between the initiation of each wave and the first reported case is consistent with observations of a delay between onset of symptoms and the diagnosis for COVID-19^22^.

Without CB, we expect the transmissibility of COVID-19 to be higher, which is confirmed by the results in Fig. 2. However, the $R_e$ should continue to rise beyond the middle of April 2020 if not for the maximum capacity imposed on the dormitory workers by our model, as explained earlier with regards to the epidemic curve without CB. The purple line in Fig. 1 gives the number of infected cases in the community. There was no substantial infection within the community initially. Large-scale infection happened only within the dormitory network after end of March. While infection spreads quickly within the dormitory network, it takes a while for the contagion to reach the community through interactions within workplaces or crowds as the probability that an individual comes into contact with a dormitory worker is very small. The virus spreads quickly within the community once it reaches a critical number and this leads to a steep rise in cases as indicated by the purple line in Fig. 1. This does not happen however when CB is implemented.

The circulation of COVID-19 within Singapore’s foreign worker dormitories exemplify an epidemic outbreak that happens within densely populated residential areas. The foreign workers constitute only $5.5\%$ of the whole population in our model, and we assume that those who reside in dormitories are poorly connected to the rest of the population. In our model, imported cases are selected uniformly at random, leaving a very small chance for dormitory residents to be an imported case. Nonetheless, epidemic outbreak is observed to occur within the dormitories in 43 out of 50 of our simulations, with the outbreak arriving earlier in some simulations relative to others. These results have led us to envisage the impact of high transmissibility in such densely populated residential areas to be far more ravaging in low-income countries where millions of people live in slums.

We have employed a SEIR multiplex network model to simulate the progression of the COVID-19 pandemic in Singapore. We have demonstrated the utility of our model in evaluating the efficacy of Circuit Breaker in containing the spread of the virus. With the Circuit Breaker ending on 1 June 2020, there are uncertainties on the strategic approaches to lift the various imposed social restriction orders. We envisage that our empirically-fitted SEIR multiplex network model could play the role of simulating potential strategies with differing level of social interaction limitations, with the purpose of determining the right amount of social-economic functions that can be restarted in Singapore without causing any harm to the successful containment effort already achieved on COVID-19.

As such, we have explored the use of our model to simulate a gradual relaxation of social restrictions from 1 June 2020 to 30 June 2020. Here, we assume that there is no imported cases during the period under study. We further assume a full restoration of inter-household social connections while intra-household connections remain unchanged as in CB. The reopening of businesses and workplaces in Singapore after the Circuit Breaker is known to occur in phases. Here, for simplicity, we consider $30\%$ of the non-essential workplaces to become socially connected again within the workplace network, with $15\%$ of workplaces for essential services to remain status quo throughout the month. As a result of the returning of the workforce to their workplaces, we increase the crowd frequency to $f_c = 600$. We also increase $f_g = 400$, but with a smaller average group size of $N_g = 25$. Finally, we assume the social connection within the dormitory network to restore to $50\%$. Note that temporary isolation facilities such as factory-converted dormitories have been employed to house foreign workers during the CB period, which minimizes social contact. Some of the workers are allowed to go back to their premises after June 2020^23^ depending on their swap test results. We thus restore $50\%$ of the social contact instead of $25\%$ after the CB. A dormitory room houses typically 12 to 16 workers before the CB. The number reduced to about 10 after the CB^24^.

**Figure 3:**
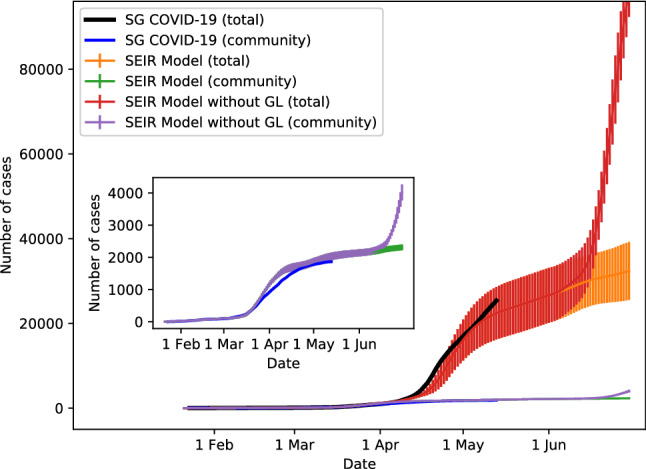
Epidemic curves for the spreading of COVID-19 in Singapore. The curves are shown for the real data, and for the simulated dynamics with and without implementation of the Gradual Lifting (GL) measures. The inset shows an enlarged view of the spreading of COVID-19 within Singapore’s community. Standard deviation is shown as vertical line for the simulated dynamics.

**Figure 4:**
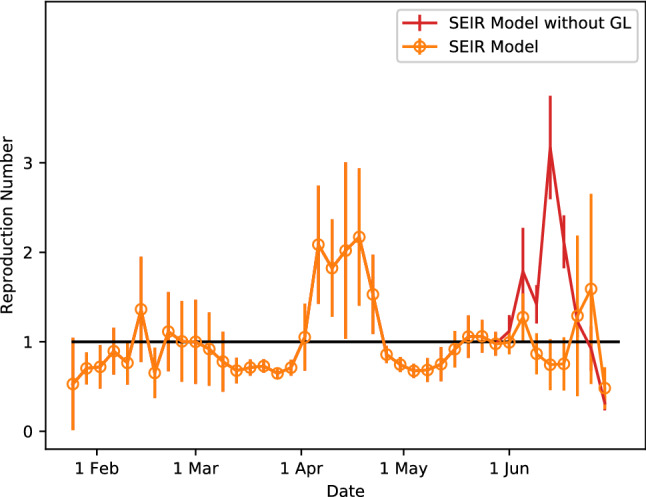
The dynamical evolution of the reproduction number $R_e$ derived from the SEIR multiplex model with and without Gradual Lifting (GL) measures. Note that the two very small waves of COVID-19 transmission at 14 Feb 2020 and 22 Feb 2020 discussed in the main text (see Fig. 2) are slightly shifted in this plot due to the large statistical fluctuations.

Our simulation results based on these parameters (see Figs. 3 and 4 ) show that the strategy of gradual lifting of social restrictions will flatten the epidemic curve, while a drastic resumption of social interactions to the period of 21 Jan 2020 will lead to another large wave of COVID-19 transmission. Hence, our model indicates that prudent public health policies that avoid massive social interactions are quintessential in maintaining the current state of pandemic control. We have simulated the gradual relaxation before 1 June 2020 with a population size of $N=1,000,000$ and similar results are obtained^25^.

**Figure 5:**
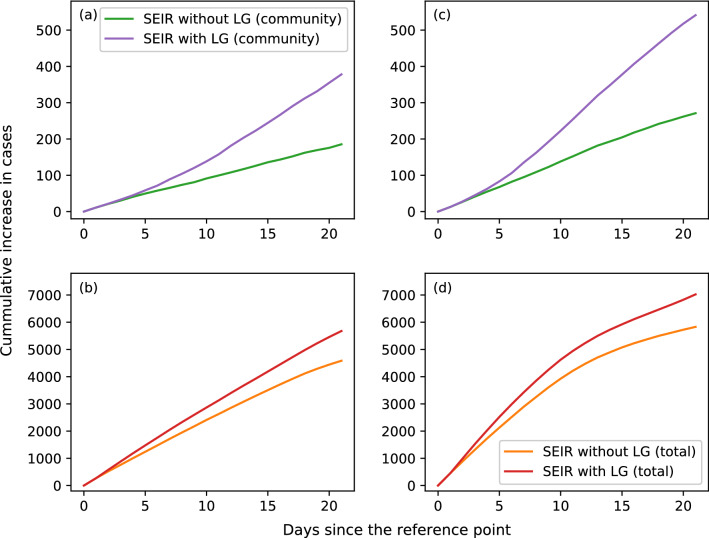
The cumulative increase in the number of new infection cases since the reference day. The two reference days are: 12 May for (**a**) and (**b**) after the outbreak has slowed down, and 21 April for (**c**) and (**d**) when the outbreak has reached its peak. The two curves in each subplot show the cumulative increase in new cases in the presence or absence of daily large-scale gathering (LG) for a population size of $N=1,000,000$. Subplots (**a**) and (**c**) illustrate the increase of new infection cases in the community, while subplots (**b**) and (**d**) indicate the total number of new infection cases.

In addition, we have used our model to study the impact of large-scale gathering at two different stages of the epidemic, namely when the outbreak reaches its peak and after the outbreak has slowed down. Specifically, we take 21 April and 12 May as two reference points and simulate the epidemic with and without the presence of large-scale gathering for an average group size of $N_g = 10,000$ in a population of $N=1,000,000$. Once the simulation reaches its reference point, we consider the scenario of (a) full social connections in household, (b) $50\%$ social connections for workplaces of non-essential services, (c) $15\%$ connections for workplaces of essential services, and (d) $50\%$ social network connections in dormitory. We set the crowd frequency to $f_c = 300$ with $N_c = 50$. We have also set $f_g$ to 1 for each of the 21 simulated days in a society with large-scale gatherings. We then compare the accumulated increase in the number of new cases since the reference points (see Fig. 5). In Fig. 5a where there is no large-scale gathering for the reference point of 12 May, 185 new cases are observed in the community at the end of the simulated period. The number doubles to 378 at the community level when large-scale gathering is enabled in our model. At the population level, 4, 586 new cases are observed, with the number increases to 5, 678 when large-scale gathering is held everyday (see Fig. 5b). Similar results are obtained when gatherings are held beyond the peak of the outbreak on 21 Apr (see Fig. 5c,d). We see an increase in community cases from 271 to 541, whereas the total number of new infection cases increases from 5, 830 to 7, 025 when large-scale gathering happens everyday for a period of 3 weeks.

## Discussion

More often than not, a model needs to be suitably augmented to capture the greater complexity exhibited in real-world physical scenarios. Our model incorporates the crucial components of social interactions through the paradigm of a multiplex of social connections to give an accurate depiction of the actual transmission scenarios of COVID-19. A multiplex-network representation of social contacts enables greater flexibility in the modelling of different types of social interaction for the simulations and evaluations of social exclusion strategies for epidemic control. In addition, a proper categorization of social entities through multiplex network provides a formal mathematical structure that is well-suited for a detailed study of epidemic spreading within the different social communities.

Note however that the model is useful only for short-term forecast and planning as the pandemic also depends on a number of unforeseen events such as a sudden increase in imported cases or a change in government policy. The exact impacts of such changes on social interactions are unclear. Frequent calibration with updated data is thus necessary to capture the evolution of social interaction pattern and dynamics of the pandemic.

## Methods

### Reproduction number

The basic reproduction number $R_0$, which is defined as the number of secondary infections caused by an infected individual, is a closely monitored measure that reveals the current state of the epidemic outbreak. It serves to inform government officials and epidemiologists who are tasked to contain the pandemic whether an outbreak is imminent. If the reproduction number is above 1, the infectious cases increase and the occurrence of an outbreak is expected. On the other hand, if $R_0$ is below 1, the number of infectious cases diminishes and the epidemic is well-contained. $R_0$ changes dynamically as the virus spreads to different parts of the population in consequence of social interactions among individuals and communities. The introduction of interventions such as travel ban and social distancing have the positive effect of lowering the value of $R_0$. Indeed, the reproduction number was found to decline from 2.35 to 1.05 after Wuhan was locked down in late January^26^.

Two common methods to estimate the reproduction number are the maximum likelihood estimator and the branching process estimator^27^. Here, we adopt the branching process estimator to evaluate the effective reproduction number $R_e$, instead of $R_0$ since the population become partially susceptible after the outbreak begins. First, we group a time series of daily number of new cases $n=\{n_0, n_1, n_2, \cdots , n_T\}$ of length $T+1$ into *m* generations, where each generation consists of $T_e$ number of data points. We then form the set:

1 $$\begin{aligned} n^* = \left\{ n_0, \sum _{t=1}^{T_e} n_t, \sum _{t=T_e+1}^{2T_e} n_t, \cdots , \sum _{t=(m-1)T_e+1}^{m T_e} n_t \right\} \,. \end{aligned}$$

Note that the mean exposed time $T_e$ gives the time between infections in consecutive generations or the serial interval. As we are concerned with an evolving $R_e$, we estimate $R_e$ for each generation *i* as:

2 $$\begin{aligned} R_e(i) = \frac{n_i^*}{n_{i-1}^*} \,. \end{aligned}$$

New cases are reported and included in an epidemic database after the infected individuals are tested positive. There is however no simple or direct relationship between the number of reported cases and the number of actual new infectious cases. In Ref.^28^, a linear regression model was used to impute the missing onset date of infection taking into account the reporting delay distribution. Here, we deduce the onset date with our SEIR model. Specifically, we simulate $M=50$ realizations of the pandemic with our SEIR model and select $20\%$ of them which best-fit the epidemic curve under study. The number of new infectious cases are then deduced from these realizations.

### SEIR model with multiplex networks

In our simulation, we use an effective population size of $N=2,000,000$ instead of the actual Singapore’s population size of 5.6 million in 2020. This assumption is reasonable because the number of infected cases $\sum _{t=0}^T n_t$ is relatively small compared to the actual population size. Hence, it is feasible to assume that a large proportion of the population has a vanishing chance of coming into contact with any infectious individual during the period under study. This assumption has the advantage of reducing the time of the simulation.

In a similar vein, we have assumed that 110, 000 foreign workers stay in the dormitories instead of the actual number of 323, 000^30^. Dormitories in Singapore are of different sizes, the largest sites house at least 1, 000 people^30^. For simplicity, we have assumed that each dormitory accommodates 1, 000 individuals in our model. As a result, our dormitory network shall consist of 110 communities. Each floor in a dormitory typically houses 120 to 180 workers^23,29^. After calibration (see the supplementary material for details), we set the average degree of the dormitory network to be $k_d=60.15$. Most of the network connections are within the community of a dormitory, with each individual in a dormitory connected to about 0.15 other individual in the other dormitory.

The rest of the population of size 1, 890, 000 are assumed to stay in households of average size $S_h=2.5$. A sample household size is drawn randomly from a Poisson distribution with a mean of $\lambda =2.5$. Members in the same household are socially connected with a probability of 0.95. Moreover, we assume that on average each individual in the household network is socially connected to 0.4 other individuals in another household.

Next, we model economically active individuals in the household network by connecting $45\%$ of them within the workplace network. Note that these individuals include students “working” in schools. Also, $90\%$ of the individuals in the dormitory network are included in the workplace network. Furthermore, workers who stay in dormitories have higher chance to work in the same workplace. The workplace network is assumed to be modular in nature with an average community size of $S_w =4$. The size of a workplace is drawn randomly from a gamma distribution with a shape parameter of 2 and a scale parameter of 2. Members within the same workplace are socially connected with a probability of 0.7, while the social connection between workers of different workplace is set at 0.08. In addition, we have introduced a small number of inter-network household-to-workplace links that connect non-working household individuals with workplace individuals. Similar household-to-dormitory inter-network links are also inserted within our multiplex network model.

In the case of our temporal crowd network, we have set $f_c=1,000$ and $N_c=50$ for each simulated day. The parameters for the temporal social gathering network are: $f_g=400$, $N_g = 50$ and $k_g=8$.

Our simulation begins with the scenario in 21 Jan 2020, two days before the first reported case of COVID-19 in Singapore, and end on 13 May 2020. Singapore implemented Circuit Breaker (CB) on 7 April 2020 to restrict social interactions among its residents. After the CB, we set $f_g=0$ and $f_c=400$. We turn off social connections in $85\%$ of the workplaces, with the remaining $15\%$ continue to be functional with essential workers physically at work. We have also turned off social connections between workplaces. On the other hand, social connections within the households remain unchanged, albeit we turn off $95\%$ of the social connections between households while maintaining $5\%$ inter-household connections to represent violations of stay-home measures. To model Singapore’s government effort in revamping the living conditions of the foreign workers, we reduce the social connections within dormitories by cutting down $25\%$ of the connections every 5 days until there are $25\%$ of the connections left. Finally, we set *p* to 0.1^31^ and $T_e$ to 4. $T_i$ is set to 3 for the first 30 days from 21 Jan 2020 to reflect the longer delay from disease onset to case report at the early phase of the pandemic. It is adjusted to a value of 2 afterward. The values of the parameters are either judiciously drawn from the literature or they are calibrated from the real-data.

### Calibration

To compare the results of simulations with the data, we calculate the mean absolute error $e_{L_1}$ which is defined as follows:

3 $$\begin{aligned} e_{L_1}=\frac{1}{T} \sum _{t=1}^T \left| N_{total}^M(t)-N_{total}^D(t) \right| \,, \end{aligned}$$

where $N_{total}^M(t)$ is the accumulated number of infected cases at time *t* simulated from the model while $N_{total}^D(t)$ is the accumulated number of infected cases at time *t* given by the data. In other words, we examine the mean of the first order error over the time span *T*.

## Supplementary Information

Supplementary Information.

## References

[1] Claeson M, Hanson S (2021). COVID-19 and the Swedish enigma. Lancet.

[2] Hellewell J (2020). Feasibility of controlling COVID-19 outbreaks by isolation of cases and contacts. Lancet Glob. Health.

[3] Arenas, A., Cota, W., & Gómez-Gardeñes, J., *et al.* A mathematical model for the spatiotemporal epidemic spreading of COVID19. *MedRxiv* 2020.03.21.20040022 (2020).

[4] Chinazzi M (2020). The effect of travel restrictions on the spread of the 2019 novel coronavirus (COVID-19) outbreak. Science.

[5] Domenico, L. D., Pullano, G., Sabbatini, C. E., Boëlle, P. Y. & Colizza, V. Expected impact of reopening schools after lockdown on COVID-19 epidemic in l̂le-de-France. *MedRxiv* 2020.05.08.20095521 (2020).

[6] Luo, J. Predictive modeling of COVID-19. *White Paper 2020*.https://ddi.sutd.edu.sg/ (accessed May 20, 2020).

[7] Pung R (2020). Investigation of three clusters of COVID-19 in Singapore: Implications for surveillance and response measures. Lancet.

[8] Estrada E (2020). COVID-19 and SARS-CoV-2. Modeling the present, looking at the future. Phys. Rep..

[9] Li, M. L., Bouardi, H. T., Lami, O. S., Trikalinos, T. A., Trichakis, N. K. & Bertsimas, D. Forecasting COVID-19 and analyzing the effect of government interventions. *MedRxiv* 2020.06.23.20138693 (2020).

[10] Statistical Machine Learning Lab of University of California at Los Angeles, COVID-19 Information Site. https://www.covid19.uclaml.org/ (accessed May 20, 2020).

[11] Kivelä M (2014). Multilayer networks. J. Complex Netw..

[12] Holme P, Saramäki J (2012). Temporal networks. Phys. Rep..

[13] Chen X (2018). Suppressing epidemic spreading in multiplex networks with social-support. New J. Phys..

[14] Wu Q, Lou Y, Zhu W (2016). Epidemic outbreak for an SIS model in multiplex networks with immunization. Math. Biosci..

[15] Marceau V, Noël P, Hébert-Dufresne L, Allard A, Dubé LJ (2011). Modeling the dynamical interaction between epidemics on overlay networks. Phys. Rev. E.

[16] Dickison M, Havlin S, Stanley HE (2012). Epidemics on interconnected networks. Phys. Rev. E.

[17] Liu QH, Xiong X, Zhang Q, Perra N (2018). Epidemic spreading on time-varying multiplex networks. Phys. Rev. E.

[18] Di, L., Gu, Y., Qian, G. & Yuan, G. X. A dynamic epidemic model for rumor spread in multiplex network with numerical analysis. ArXiv:2003.00144v1 (2020).

[19] Li, T. Simulating the spread of epidemics in China on the multi-layer transportation network: Beyond the coronavirus in Wuhan. ArXiv:2002.12280v1 (2020).

[20] Kupferschmidt K (2020). The lockdowns worked: But what comes next?. Science.

[21] Singapore Ministry of Health. https://www.moh.gov.sg/covid-19.

[22] Tariq A (2020). Real-time monitoring the transmission protocol of COVID-19 in Singapore, March 2020. BMC Med..

[23] Meah, N. Cleaner but still crowded, say recovered foreign workers returning to ‘virus-free’ dorms. *Today* 13 June 2020. https://www.todayonline.com/singapore/cleaner-still-overcrowded-say-recovered-foreign-workers-returning-virus-free-dorms(accessed Aug 03, 2020).

[24] Nadarajan, R. New dorms with ’better standards’ to be built for 100,000 foreign workers in coming years: Lawrence Wong. *Today* 1 June 2020. https://www.todayonline.com/singapore/new-dorms-better-standards-be-built-100000-foreign-workers-coming-years-lawrence-wong (accessed Aug 03, 2020).

[25] Chung, N. N. & Chew, L. Y. Modelling singapore covid-19 pandemic with a SEIR multiplex network model. *MedRxiv* 2020.05.31.20118372 (2020). 10.1038/s41598-021-89515-7 PMC8115043 33980920

[26] Kucharski AJ (2020). Early dynamics of transmission and control of COVID-19: A mathematical modelling study. Lancet Infect. Dis..

[27] White LF, Pagano M (2008). A likelihood-based method for real-time estimation of the serial interval and reproductive number of an epidemic. Stat. Med..

[28] White LF (2009). Estimation of the reproductive number and the serial interval in early phase of the 2009 influenza A/H1N1 pandemic in the USA. Influenza Respir. Viruses.

[29] Lim, J. Coronavirus: Workers describe crowded, cramped living conditions at dormitory gazetted as isolation area. *The Straits Times* 6 April 2020. https://www.straitstimes.com/singapore/manpower/workers-describe-crowded-cramped-living-conditions (accessed 4 Feb 2021).

[30] Ng, K. G. Over 80% of dorm residents coronavirus-free, 95 more dormitories cleared of Covid-19: MOM. *The Straits Times* 30 July 2020. https://www.straitstimes.com/singapore/over-80-of-dorm-residents-coronavirus-free-95-more-dormitories-cleared-of-covid-19-mom.

[31] Fang Y, Nie Y, Penny M (2020). Transmission dynamics of the COVID-19 outbreak and effectiveness of government interventions: A data-driven analysis. J. Med. Virol..

