# Supplementary material for: Modelling Singapore COVID-19 pandemic with a SEIR multiplex network model
Source: Sci Rep. 2021 May 12;11:10122. doi: 10.1038/s41598-021-89515-7 (PMC8115043; doi:10.1038/s41598-021-89515-7)
Supplement: Supplementary file 1 — Supplementary Information. [file 41598_2021_89515_MOESM1_ESM.pdf]

Modelling Singapore COVID-19 pandemic with a SEIR multiplex network model

N. N. Chung[1,*] **and L. Y. Chew**[2,3,4,+]

[1]Centre for University Core, Singapore University of Social Sciences, Singapore 599494.
[2]School of Physical & Mathematical Sciences, Nanyang Technological University, Singapore 637371.
[3]Data Science & Artificial Intelligence Research Centre, Nanyang Technological University, Singapore 639798.
[4]Complexity Institute, Nanyang Technological University, Singapore 637723.
[*]nnchung@suss.edu.sg
[+]lockyue@ntu.edu.sg

Supplementary Materials

In this supplementary material, we provide additional results and information.

Social Network

We simulate the dynamics of the epidemic outbreak in Singapore using the Erdős-Rényi (ER) random network and the scale-free (SF) network. Here, we do not capture the complex interaction of modern society, instead, social contacts are represented by simple ER random networks and scale-free networks with and without community structure. For both cases, the parameters used are $N = 1,000,000$, $p = 0.1$, $T_e = 4$ and $T_i = 2$. We have set the average degree (or number of connections) of the ER random network and the SF network at $k = 8$ and $k = 2$ respectively. Note that we have used a scale-free network of a smaller average degree because epidemic spreading is faster in SF network compared to ER network of the same average degree. For each case, we simulate 50 realizations and select 20% of them which best-fit the epidemic curve under study.

The simulated epidemic curves with ER networks are shown in Fig. S1. We observe that the curves cannot accurately depict the dynamics of epidemic outbreak in Singapore. In particular, the simulated outbreak begins much earlier than the actual outbreak, and saturates at a much lower number of infection cases compared to the actual outbreak. This result holds for both epidemic spreading in ER network with and without community structures. The corresponding $R_e(t)$ deduced from the simulation is shown in Fig. S2. The dynamics is very different from those obtained from the SEIR model with multiplex network. Here, R_e is maintained almost constant at around 1.2 from 21 Jan to 5 April before dropping abruptly to a value around 0.4.

The simulated epidemic curves with SF networks are shown in Fig. S3. Again, the best-fit epidemic curve cannot accurately model the dynamics of the actual outbreak. The simulated outbreak happens much earlier than the actual outbreak. It picks up quickly and then saturates within a short time span. The consequence is a single peak in the corresponding $R_e(t)$ which is shown in Fig. S4.

Calibration

Here, we take the calibration of the average degree of the dormitory network as an illustration of the manner we performed the calibration. We simulated epidemic spreading with a dormitory network of intra-community degree that ranges from 40 to 80. We compared the dynamics with the data to yield an average intra-community degree that gives the smallest difference between them. In addition, we tune the inter-community connection within the network to obtain a minimum mean absolute error between the simulation results and data. These results are illustrated in Figure S5. With these calibrations, we set the average degree of the dormitory network to be $k_d = 60.15$.

Additional details for the study of Singapore COVID-19 pandemic

In Fig. S6, we show the results of all 50 simulations of Fig. 1 of the main text. The simulation employs the SEIR multiplex network to investigate Singapore's COVID-19 pandemic for the period 21 Jan 2020 to 13 May 2020. In Fig. S7, we show the number of infection cases within the dormitory network. Finally, we show in Fig. S8 the dynamical effective reproductive number for the propagation of the COVID-19 epidemic in Singapore if the Circuit Breaker measures are not lifted until 30 June 2020. We observe R_e to fluctuate around 1 from mid-May to early June. The fluctuation in R_e reflects the fluctuation of the number of reported cases in Singapore's COVID-19 database. If the lifting were delayed until end of June, we found that R_e could be further reduced to around 0.6 which is lower compared to the value of about 0.8 when CB is lifted on 1 June 2020.

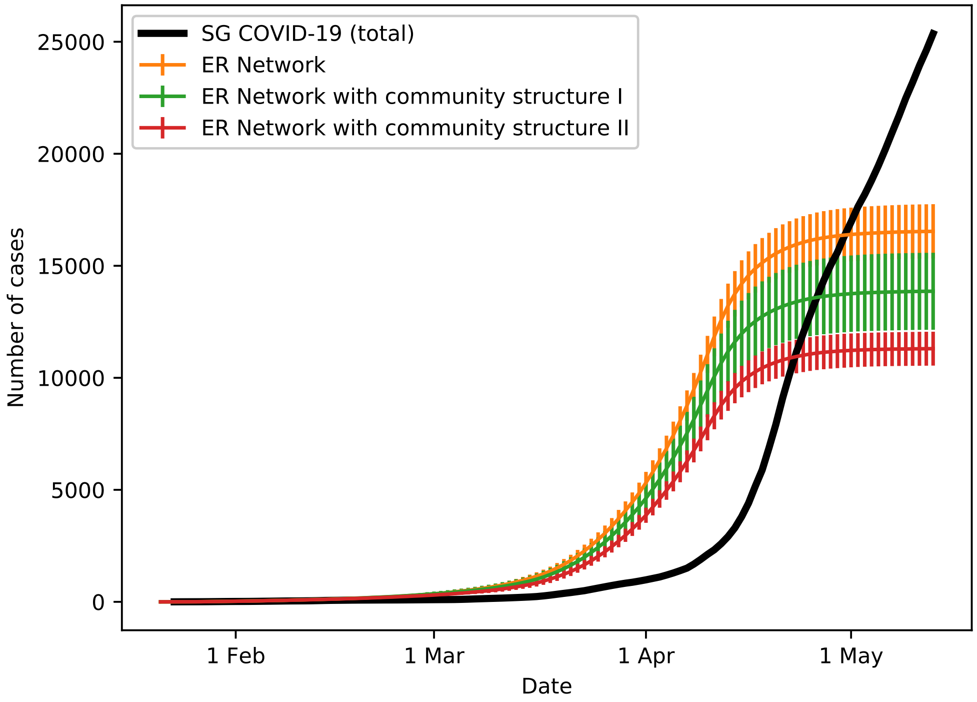

Figure S1. Epidemic curves simulated with SEIR model and Erdős-Rényi (ER) random network for the spreading of COVID-19 in Singapore. The curves are shown for the real data, and for the simulated dynamics with ER network with and without community structure. Community structure I and community structure II are created by implementing $8N$ and $16N$ link rewirings to connect nodes within the same community of the ER random network respectively. Standard deviation is shown as vertical line for the simulated dynamics.

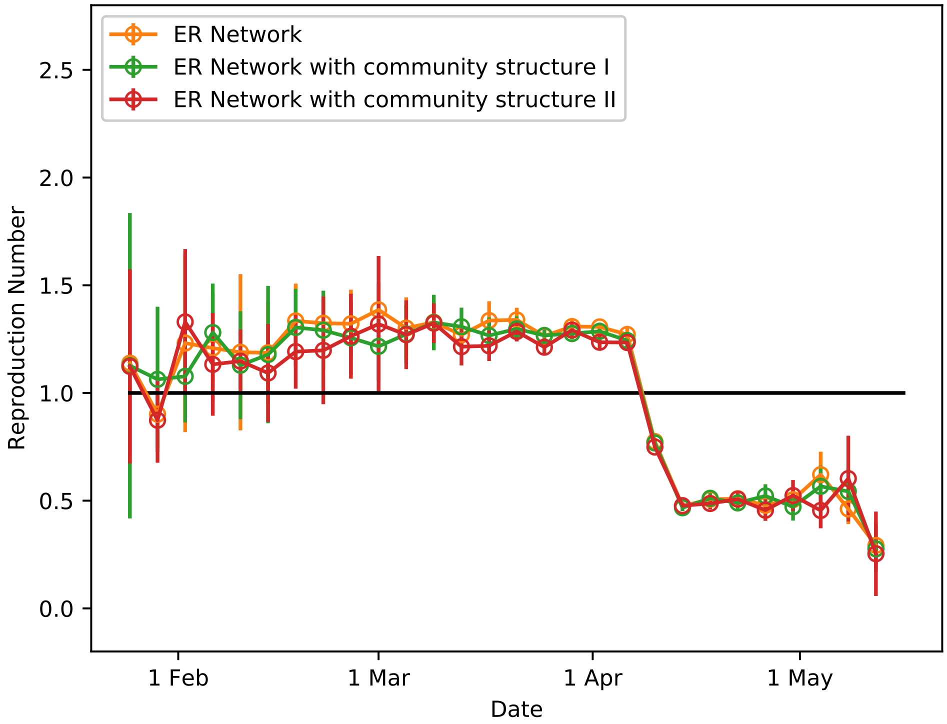

Figure S2. The dynamical evolution of the reproduction number R_e derived from the SEIR model with Erdős-Rényi network.

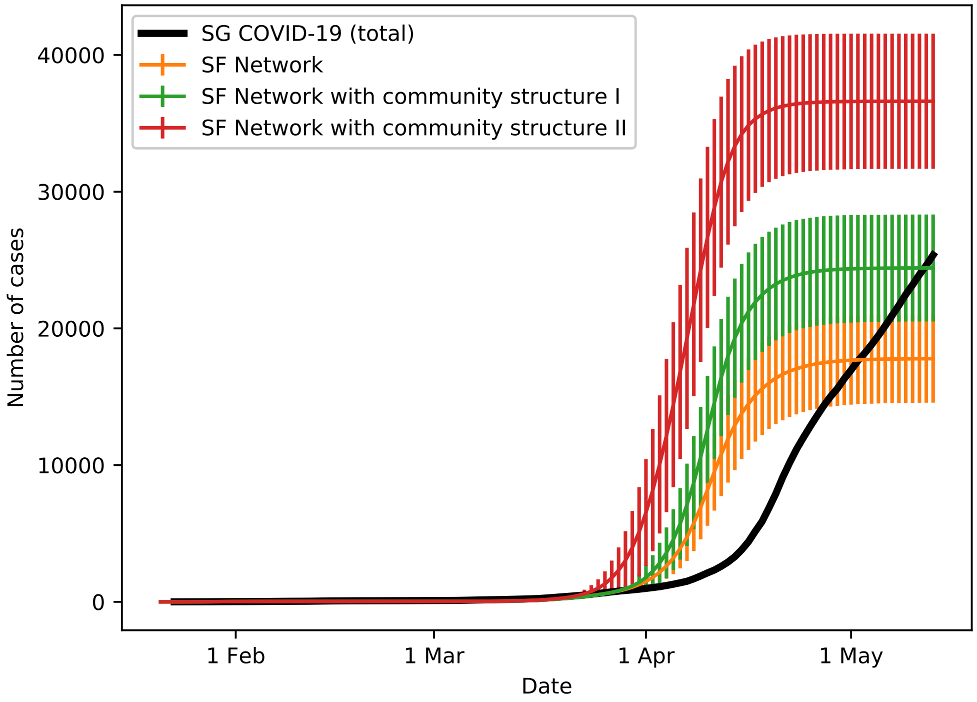

Figure S3. Epidemic curves simulated with SEIR model and scale-free (SF) network for the spreading of COVID-19 in Singapore. The curves are shown for the real data, and for the simulated dynamics with SF network with and without community structure. Community structure I and community structure II are created by implementing 2N and 8N link rewirings to connect nodes within the same community of the SF network respectively. Standard deviation is shown as vertical line for the simulated dynamics.

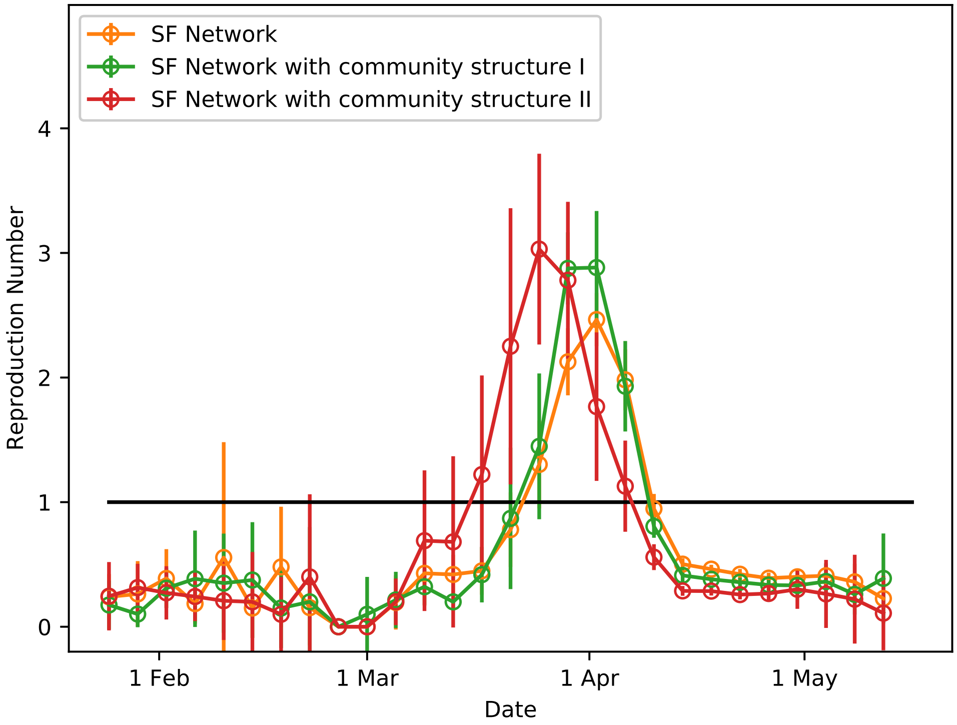

Figure S4. The dynamical evolution of the reproduction number R_e derived from the SEIR model with scale-free network.

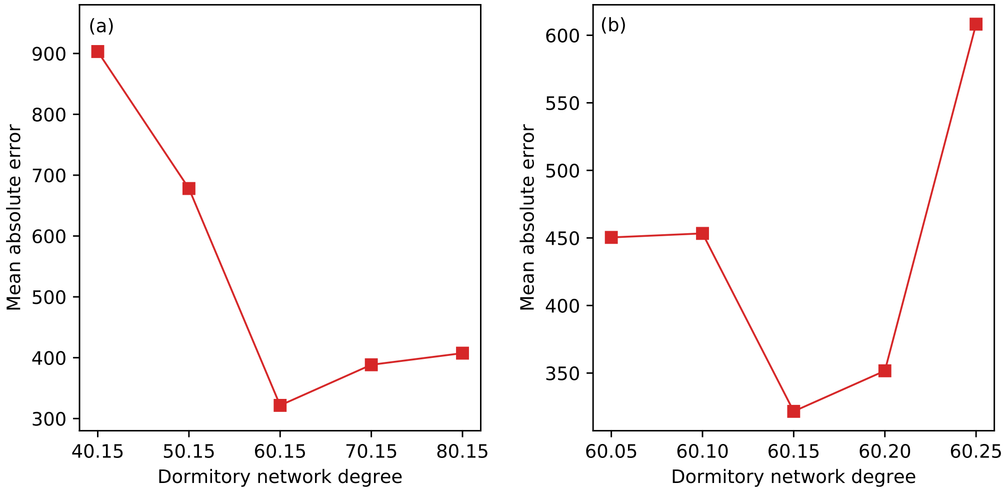

Figure S5. Calibration of the average degree of the dormitory network through (a) intra-community connections and (b) inter-community connections.

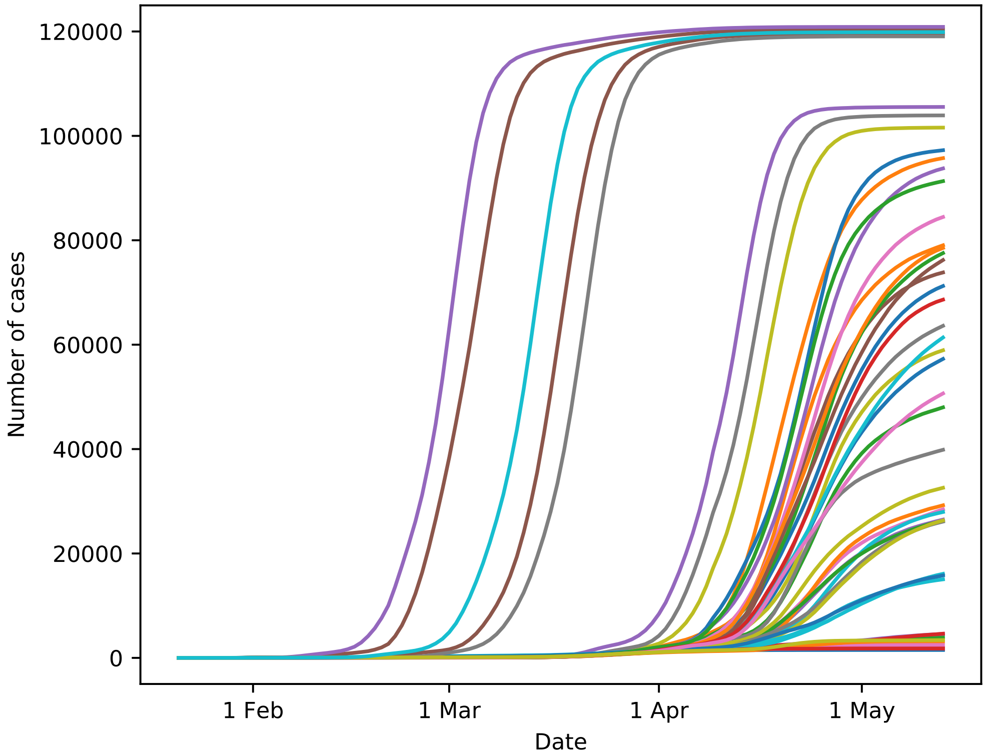

Figure S6. The 50 realizations simulated for Singapore's COVID-19 pandemic using the SEIR multiplex network model. Note that 20% of these realizations which best-fit the real-world epidemic curve under study is used to model the pandemic in Fig. 1 of the main text.

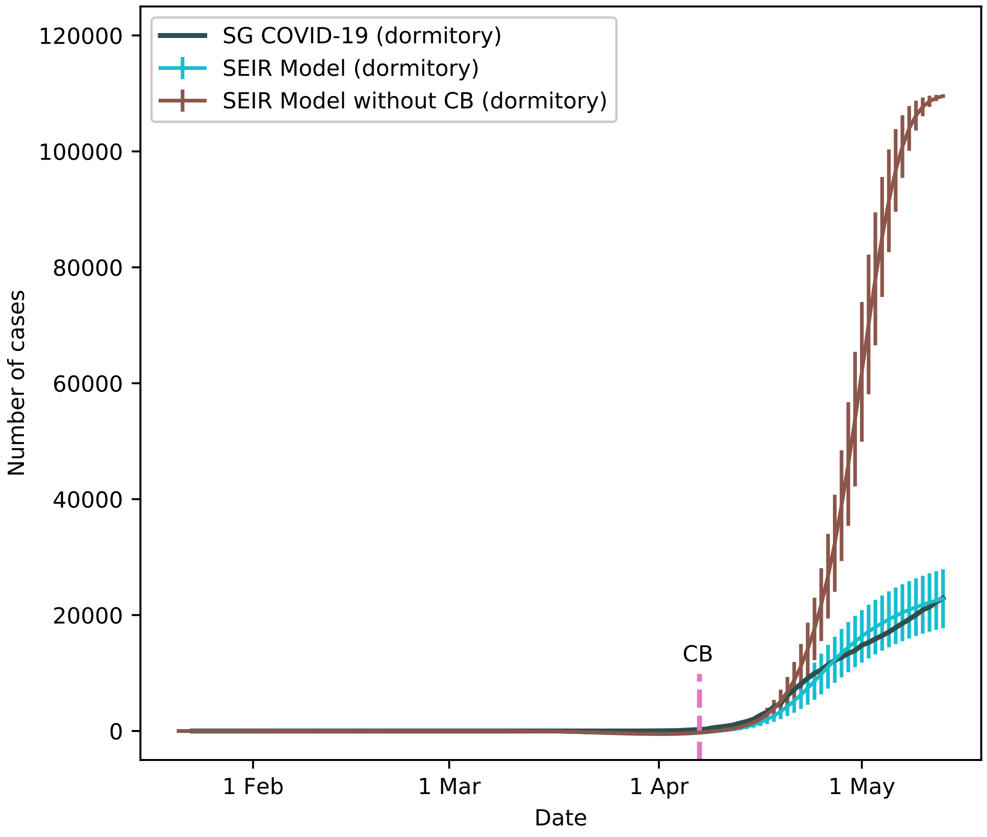

Figure S7. Epidemic curves illustrating the spread of COVID-19 in dormitories in Singapore. The curves are shown for real data, and for simulated dynamics with and without implementation of the Circuit Breaker (CB) measures. Standard deviation is shown as vertical line for the simulated dynamics. Note that dashed vertical lines in both plots mark the beginning of CB.

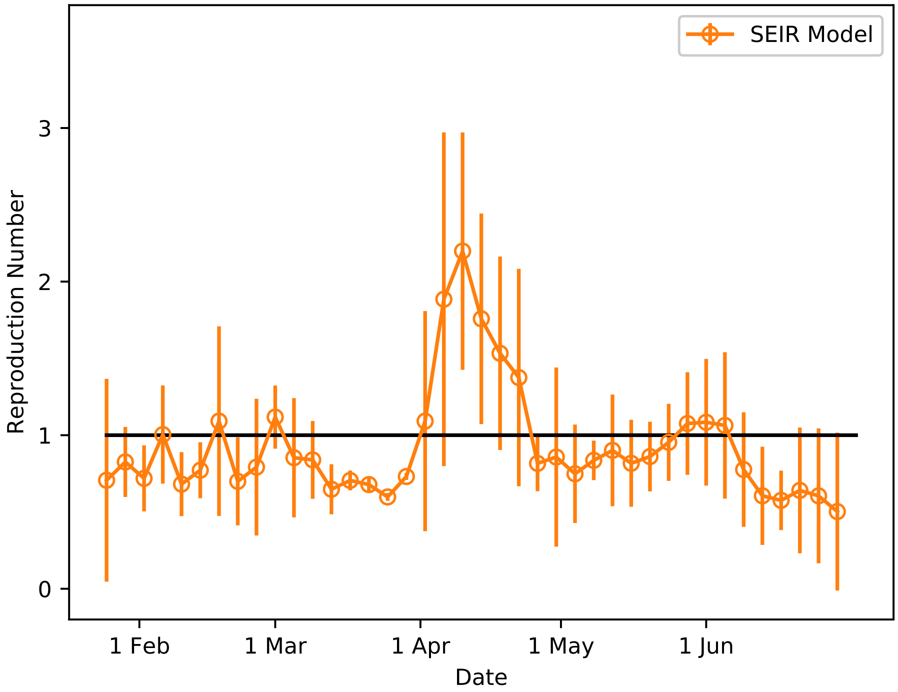

Figure S8. The dynamical evolution of the reproduction number R_e derived from the SEIR multiplex network model from 21 Jan 2020 to 30 June 2020 by assuming that CB is not lifted till 30 June 2020.
